# Supplementary material for: Comprehensive Comparison of 22C3 and SP263 PD-L1 Expression in Non-Small-Cell Lung Cancer Using Routine Clinical and Conditioned Archives
Source: Cancers (Basel). 2022 Jun 27;14(13):3138. doi: 10.3390/cancers14133138 (PMC9265108; doi:10.3390/cancers14133138)
Supplement: Supplementary file 1 [file cancers-14-03138-s001.zip › cancers-1780901-supplementary.pdf]

Table S1: Clinicopathologic characteristics of 314 FFPE archives

	No. of Patient (%)	
Age		
Range	46 - 80	
Median	66	
Gender		
Male	169	(53.8)
Female	145	(46.2)
Smoking		
Non-smoker	134	(42.7)
Smoker	180	(57.3)
Tumor size (cm)		
3	144	(45.9)
>3	170	(54.1)
Tumor Type		
Adenocarcinoma	210	(66.8)
Squamous cell carcinoma	104	(33.2)
T classification		
T1	105	(33.4)
T2	170	(54.1)
T3	39	(12.5)
Stage		
I	103	(32.8)
II	165	(52.5)
III	46	(14.7)
Surgical procedure		
Lobectomy	75	(94.4)
Pneumonectomy	4	(5.6)

FFPE, formalin-fixed paraffin-embedded
